# Dental Colleges—Commencements

**Published:** 1891-08

**Authors:** 


### EDITORIAL.

### Dental Colleges—Commencements.

All the dental colleges of this country, numbering about thirty-seven, have concluded their work for the term of 1890 and 1891.

A list of the graduates of nearly all these, for the term just closed, will be found in the DENTAL REGISTER of the months of May, July and August, to which ready reference may be made by any desiring to do so. From thirty of these the degree of Doctor of Dental Surgery was conferred on 1,164 persons; this making an average of thirty-eight and a fraction for each college; this aproximately is about one-twelfth of the number of dentists practicing in this country. This is a much larger number than has been graduated in any former year. A little consideration of this question will, we think, quiet the fears so often expressed, "That the dental profession is soon to be overcrowded with practitioners." We think it not wide of the mark to say that every year one in every fifteen of the practitioners will leave its ranks by death, age, incapacity, or for the prosecution of other business; doubtless, at least one in twenty of the recent graduates from our colleges, will for want of adaptation or some other cause, never enter fully into the practice. If these estimates are proximately true, the additions made by the colleges will not more than keep up the number to its present point. Owing to the legal regulations, and the change in public sentiment, there are very few additions to the profession except through the colleges. It is but a few years since the large proportion of those taking up the practice of dentistry did so in some other way than through the colleges; that is now practically stopped, and it is not difficult to estimate with some degree of accurateness the number entering the profession annually. Whether the number of graduates is to be kept up to the present standard, or increased for the next few years, is a question that each one will decide for himself. The probabilities are, however, that for the next two year, and probably the year following, as large, or a larger number will be graduated from the colleges than this year; after the next two years, however, and for several years thereafter the probability is that there will not be any increase; the number will probably be less for several years after that time, at least, three or four years. The increase in numbers in the profession has, in years past, not more than kept up with the increase of population.

While it may seem that in some localities there are more dentists really than are needed, the fact is that nowhere is there an excess of the better class of operators, and in mny places there are none of the better class, and there are many communities through this country where there is no dentist of any kind. The demand everywhere is for better service. The people are becoming rapidly educated on this question, and it is very questionable whether the profession is keeping up with that demand. This education of the people will continue, the demand for better preparation will grow, and because of this condition many of those who have been practicing in a very defective way find themselves crowded out and forced to seek some other occupation.

It is to be hoped that this process of elimination and progress will continue with increased ratio from this time henceforth.

